# Irreversible and Repeatable Shape Transformation of Additively Manufactured Annular Composite Structures

**DOI:** 10.3390/ma14061383

**Published:** 2021-03-12

**Authors:** Bona Goo, Jong-Bong Kim, Dong-Gyu Ahn, Keun Park

**Affiliations:** 1 Department of Mechanical System Design Engineering, Seoul National University of Science and Technology, Seoul 01811, Korea; qhsdk96@seoultech.ac.kr; 2 Department of Mechanical and Automotive Engineering, Seoul National University of Science and Technology, Seoul 01811, Korea; jbkim@seoultech.ac.kr; 3 Department of Mechanical Engineering, Chosun University, Gwang-ju 61452, Korea; smart@chosun.ac.kr

**Keywords:** additive manufacturing, 4D printing, material extrusion, irreversible shape transformation, circumferential anisotropy

## Abstract

Four-dimensional (4D) printing is a unique application of additive manufacturing (AM) which enables additional shape transformations over time. Although 4D printing is an interesting and attractive phenomenon, it still faces several challenges before it can be used for practical applications: (i) the manufacturing cost should be competitive, and (ii) the shape transformations must have high dimensional accuracy and repeatability. In this study, an irreversible and repeatable thermoresponsive shape transformation method was developed using a material extrusion type AM process and a plain thermoplastic polymer (ABS) without a shape-memory function. Various types of annular discs were additively manufactured using printing paths programmed along a circular direction, and additional heat treatment was conducted as a thermal stimulus. The programmed circumferential anisotropy led to a unique 2D-to-3D shape transformation in response to the thermal stimulus. To obtain more predictable and repeatable shape transformation, the thermal stimulus was applied while using a geometric constraint. The relevant dimensional accuracy and repeatability of the constrained and unconstrained thermal deformations were compared. The proposed shape transformation method was further applied to AM and to the in situ assembly of a composite frame–membrane structure, where a functional membrane was integrated into a curved 3D frame without any additional assembly procedure.

## 1. Introduction

Recent advances in three-dimensional (3D) printing, also known as additive manufacturing (AM), now allow the process to be used not only for conventional prototyping but also for the direct fabrication of functional parts [1,2,3]. Four-dimensional (4D) printing is a unique application of AM that enables the shape of additively manufactured parts to be transformed over time [4]. It has various functional applications, especially for the fabrication of components where self-assembly, self-repair, or self-adaptability is required [5,6]. In 4D printing, three basic factors are necessary to achieve desirable shape transformations: stimulus-responsive materials, the relevant external stimuli, and interaction mechanisms [7]. Various combinations of stimuli and stimulus-responsive materials have been studied to develop 4D printing for smart devices or functional actuators [8,9,10].

The most widely used stimulus-responsive materials in 4D printing are so-called smart materials such as shape memory polymers (SMPs) [11]. SMPs have the unique ability to recover their original shapes when a thermal or solvent stimulus is applied [12]. Thermoresponsive SMPs undergo shape changes when a thermal stimulus higher than a critical temperature is imposed [13,14,15,16]. Solvent-responsive SMPs undergo size change in response to a specified solvent [17]. Hydrogels are the most widely used solvent-responsive SMP and swell in response to a water stimulus [18]. Both types of SMPs have been used in various 4D printing studies as a form of composite [19,20,21].

Most 4D printing studies have used a liquid photopolymer resin, solidified by photo-polymerization (PP) or material jetting (MJ) type AM methods. Material extrusion (ME) type AM has also been used for 4D printing, by printing thermoresponsive SMP filaments [22,23,24,25] or by printing multiple materials with different deformation characteristics through multiple nozzles [26,27,28,29]. Multi-material printing provided irreversible and permanent shape transformation, while the conventional 4D printing based on SMPs provided reversible and temporary shape transformation [26]. Recently, ME-based 4D printing methods were developed using programmed printing paths to produce unidirectional anisotropy [30,31,32,33] or bidirectional anisotropy [34,35,36]. These approaches enabled bending deformations of the additively manufactured specimens by applying adequate thermal stimuli. Although these studies provided versatile shape transformations, further investigations were required to improve the dimensional accuracy of the deformed shape because precise control of the transformed dimensions is essential before it can be used in an industrial application.

This study aimed to develop an industrially applicable shape transformation method for additively manufactured parts, with the following requirements: low manufacturing cost, irreversible transformation, and high dimensional accuracy with repeatability. For the low-cost AM process, we used a personal ME type 3D printer and acrylonitrile butadiene styrene (ABS) filament, which is a plain thermoplastic material without shape-memory function. To enable thermoresponsive shape transformation for this material, a circular printing path was programmed for an annular disc to generate circumferential anisotropy. The printed annular discs were then heated above the glass transition temperature, which resulted in irreversible 2D-to-3D shape transformations. The relevant circumferential anisotropy was then evaluated experimentally by observing the thermal deformation of partial annuli fabricated with various design parameters. The obtained circumferential anisotropy model was then used to analyze thermal residual stress and the resulting deformations of full annuli, as well as to explain the 2D-to-3D shape transformations of the circularly printed annular discs.

To obtain high dimensional accuracy with repeatability, the shape transformation was performed under a geometric constraint, and the resulting dimensional accuracy was compared with that of an unconstrained shape transformation. The proposed shape transformation process was further applied to AM and the in situ assembly of a composite frame–membrane structure, in which a functional membrane was inserted inside the additively manufactured annular frame. The 2D composite frame–membrane structure could then be transformed into a curved 3D shape by the constrained heat treatment process, which provided good dimensional accuracy and repeatability.

## 2. Materials and Methods

### 2.1. Materials

ABS filaments (Shenzhen ESUN Industrial Co., Ltd., Shenzhen, China) were used in the ME type AM. This material was selected because it has higher thermal shrinkage than other thermoplastic filaments such as polylactic acid (PLA) [37]. The density, glass transition temperature, and thermal expansion coefficient of this material were 1.04 g/cm^3^, 107.8 °C, and 88.2 × 10^−6^/°C, respectively. The initial diameter of the filament was 1.75 mm, and it was extruded through a hot nozzle with 0.4 mm diameter. The additively manufactured parts using this material are known to have orthotropic anisotropy in mechanical properties [38] and in apparent thermal expansion coefficients [36], as given in Table 1. Here, α*_l_* and α*_t_* denote the apparent thermal expansion coefficients of the additively manufactured parts after heat treatment, along the longitudinal and transverse printing paths, respectively.

### 2.2. Design of Annular Shapes

**Figure 1:**
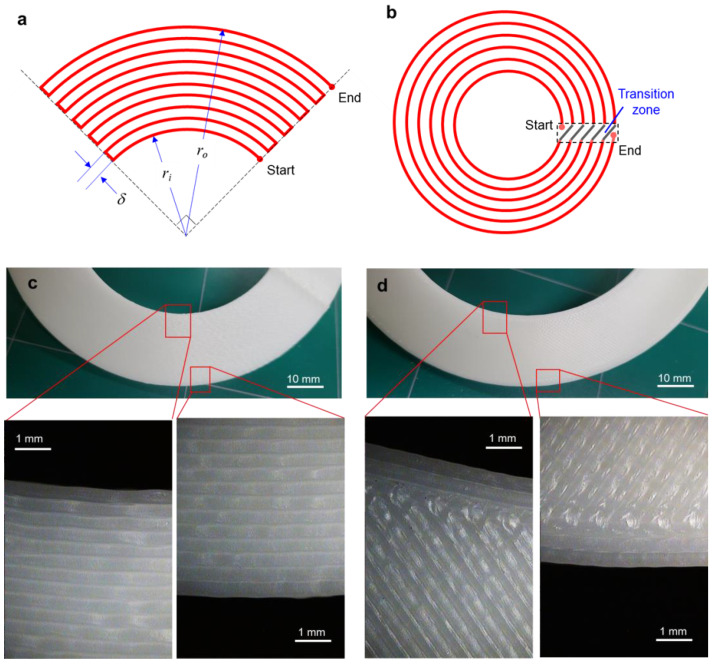
Configuration of printing paths for partial and full annuli: (**a**) circular printing path for a partial annulus; (**b**) circular printing path for a full annulus; (**c**) photographs of the printed disc with the circular printing path; (**d**) photographs of the printed disc with a typical printing path.

In ME type AM, a printing path is generally designed with alternative raster angles (for example, 45° and −45°) to minimize planar anisotropy [39]. In this study, however, the printing path was programmed in a circular direction through all layers in order to intentionally impose circumferential anisotropy. Figure 1a shows the printing path for a partial annulus (i.e., a quarter annulus) with a circular printing direction. Here, the printing path was programmed automatically with the following design parameters: the inner radius (*r_i_*), the outer radius (*r_o_*), and the printing gap (*δ*). Figure 1b shows the circular printing path for a full annulus, where the path starts at an inner pole (0° location), rotates a single turn, and is then connected to the next printing path in the transition zone. Accordingly, the printed paths form concentric circles except for the transition zone, as illustrated in Figure 1b. Figure 1c shows a photograph and microscopic images of the circular printing path, which differs from a typical printing path with 45° and −45° raster angles, as shown in Figure 1d.

### 2.3. Additive Manufacturing with Programmed Printing Paths

An ME type 3D printer (Cubicon Single, Cubicon Inc., Sungnam, Korea) was used to fabricate 2D annular discs. This printer uses an extrusion nozzle with a diameter of 0.4 mm, and the circular printing was conducted with a radial gap (*δ*) of 0.4 mm. For appropriate plasticization and stable lamination of the ABS filament, the temperatures of the nozzle, printing bed, and chamber were set to 240, 115, and 60 °C, respectively. The printing speed was set to 80 mm/s, and the layer thickness was set to 0.2 mm for the lamination of an annular disc with a thickness of 1.6 mm.

### 2.4. Heat Treatment

For the additively manufactured annular discs, heat treatment was conducted as a thermal stimulus. The printed samples were placed in an electric furnace (DHG-9070A, NeuronFit Co., Seoul, Korea), which was preheated to 150 °C. Heat treatment was conducted at this temperature for 20 min. After heat treatment, the heated samples were cooled at room temperature for 1 h.

**Figure 2:**
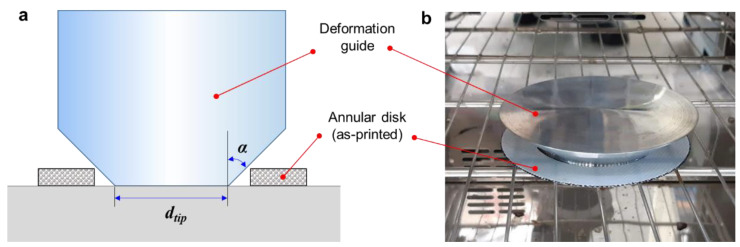
Constrained heat treatment using a tapered deformation guide: (**a**) schematic description; (**b**) photograph inside the heating chamber.

To obtain a uniform and repeatable shape transformation for the full annulus, heat treatment was also conducted while using a geometric constraint. Figure 2a illustrates the configuration of the constrained heat treatment. A tapered deformation guide was placed inside an as-printed annular disc. The deformation guide was fabricated by AA6061, and the tip diameter (*d_tip_*) and draft angle (*α*) were designed to guide the transformation of the annular sample into the desired shape. Figure 2b shows a photograph of the inside of the heating chamber of the electric furnace. Detailed results of the constrained heat treatment using this deformation guide are discussed in Section 3.4.

### 2.5. Characterization

The deformed shapes of the partial annuli after heat treatment were analyzed by 2D image processing. A digital imaging system (EOS-60d, Canon Inc., Tokyo, Japan) was used to take digital images of the deformed annuli. Digital images were taken of four samples in each case, at least 1 h after the end of heat treatment. These images were then imported into graphic software (AutoCAD^®^, Autodesk Inc., San Rafael, CA, USA) and were analyzed to graphically measure dimensional changes. The deformed shapes of the full annuli were also measured by analyzing digital images of the top and side views, which showed 3D deformations. An optical microscope (Mi-9000, Jason Electro-Tech, Seoul, Korea) was used to obtain microscopic images for the additively manufactured composite structure.

**Figure 3:**
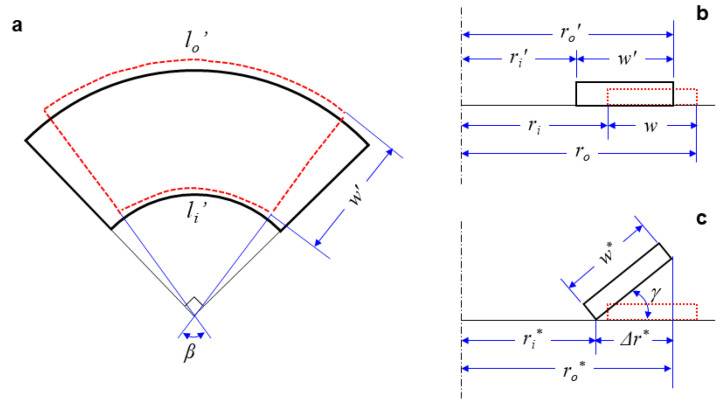
Schematic descriptions of the thermal shape transformations for (**a**) partial annulus (top view), (**b**) partial annulus (side view), and (**c**) full annulus (sectional view).

Using the measurement results, the deformation behaviors of the partial and full annuli were analytically investigated. Figure 3a,b show the deformed shapes of a partial annulus from the top and side views, respectively. Here, the red dashed lines represent deformed shapes, while the black solid lines represent the original shapes. According to the deformed geometry, the inner and outer apparent circumferential strains of the cylindrical surfaces ($\varepsilon_{\theta }^{i}$ and $\varepsilon_{\theta }^{o}$) can be expressed using the following equations [40]:

(1) $$\varepsilon_{\theta }^{i}=\frac{l_{i}{}^{\prime }-l_{i}}{l_{i}}=\frac{2\beta r_{i}{}^{\prime }}{\pi r_{i}}-1,$$

(2) $$\varepsilon_{\theta }^{o}=\frac{l_{o}{}^{\prime }-l_{o}}{l_{o}}=\frac{2\beta r_{o}{}^{\prime }}{\pi r_{o}}-1,$$

where *l* and *l*′ are the perimeters of the cylindrical surfaces before and after heat treatment, respectively. *β* is the central angle of the deformed partial annulus, and *r* and *r*′ are the radii of the cylindrical surfaces before and after heat treatment. Here, the subscripts *i* and *o* denote the inner and outer dimensions, respectively. The relevant thermal deformation and strains of the partial annuli are discussed in Section 3.1.

Figure 3c presents a sectional description of the 2D-to-3D shape transformation of a full annulus. Compared to the 2D-to-2D shape transformation of a partial annulus, the differences between the outer and inner radii (Δr) are expressed with the following equations:

(3) $$\Delta r^{\prime }=r_{o}^{\prime }-r_{i}^{\prime }=w{}^{\prime }\;(\mathrm{for}a\mathrm{partial}\mathrm{annulus}),$$

(4) $$\Delta r^{*}=r_{o}^{*}-r_{i}^{*}<w^{*}\;(\mathrm{for}a\mathrm{full}\mathrm{annulus}),$$

where *w*′ and w* denote the deformed widths of the partial and full annuli, respectively. Because the deformed width of the full annulus (w*) is larger than the radius difference (Δr*), it undergoes an unstable out-of-plane deformation, as illustrated in Figure 3c. The lift angle of the full annulus (*γ*) is then defined by the following equation, and the relevant results are discussed in Section 3.2:

(5) $$\gamma =\cos^{-1}\left(\frac{r_{o}^{*}-r_{i}^{*}}{w^{*}}\right).$$

## 3. Results and Discussion

### 3.1. Thermal Deformation of the Partial Annulus

**Figure 4:**
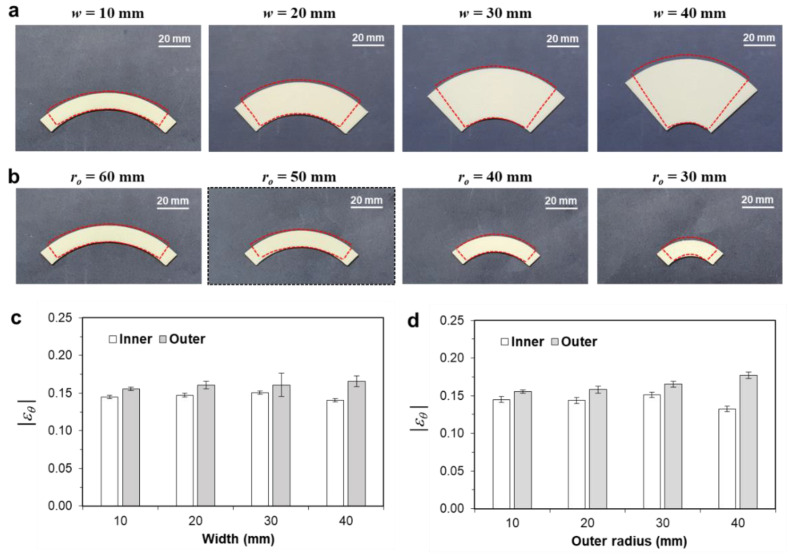
Comparison thermal deformation results of the partial annulus frame: (**a**) DEFORMED shapes with various annulus widths (*r_o_ =* 60 mm); (**b**) deformed shapes with various outer radii (*w* = 10 mm); (**c**) comparison of the circumferential strains (*ε_θ_*) with various annulus widths; (**d**) comparison of the circumferential strains (*ε_θ_*) with various outer radii. Here, the strain values are expressed as absolute values.

Figure 4a shows the deformed shapes of the partial annuli with various annulus widths (*w*). Here, the outer radius (*r_o_*) was set to 60 mm, and the width was varied from 10 to 40 mm. In all figures, the colored annuli represent the as-printed samples, and the dashed lines represent the deformed shapes after heat treatment. Overall, these annuli showed significant reductions along the circumferential direction, while exhibiting slight increases along the radial direction.

These size changes can be explained by the anisotropic residual stress introduced by the ME type 3D printing. Because a thick filament (i.e., 1.75 mm in diameter) is extruded through a thin nozzle (i.e., 0.4 mm in diameter), the printed part becomes highly oriented along the circumferential direction. The circumferential direction is under tensile residual stress, whereas the radial direction is under compressive residual stress. The relaxation of this residual stress causes anisotropic thermal deformation, which results in contraction along the circumferential direction and elongation along the radial direction. Figure 4b shows the deformed shapes of the partial annuli with various outer radii (*r_o_*). Here, the annulus width was set to 10 mm, and the outer radius was varied from 60 to 30 mm. The overall trends of significant circumferential contraction and slight radial elongation are similar to the previous results in Figure 4a.

To investigate the deformation behaviors in more detail, the apparent circumferential strain (*ε_θ_*) was calculated using Equations (1) and (2) for the inner and outer circumferential surfaces. Figure 4c,d compare the circumferential strains of the inner and outer surfaces ($\varepsilon_{\theta }^{i}$ and $\varepsilon_{\theta }^{o}$) with various annular widths and outer radii, respectively. Overall, the calculated circumferential strains were near −0.15, which was smaller than the apparent longitudinally strain of a rectangular bar (−0.196), as given in Table 1 [30]. Moreover, the circumferential strain of the outer surface ($\varepsilon_{\theta }^{o}$) was higher than that of the inner surface ($\varepsilon_{\theta }^{i}$). The difference between the two strain components was higher as the width increased or as the outer radius decreased.

Table 2 and Table 3 quantitatively compare the resulting strain components for the circumferential (*ε_θ_*), radial (*ε_r_*), and thickness (*ε_z_*) directions. Here, the superscripts *i* and *o* denote the inner and outer surfaces, respectively. It was observed that the calculated strains components had negative values along the printing direction (i.e., the circumferential direction) and positive values along the lateral direction (i.e., the radial and thickness directions) because of the residual stress in the additively manufactured parts. The strain along the thickness direction (*ε_z_*) was much larger than that along the radial direction (*ε_r_*) because the layer thickness was set to 0.2 mm, which was half the size of the nozzle diameter (0.4 mm).

### 3.2. Thermal Deformation of the Full Annulus

**Figure 5:**
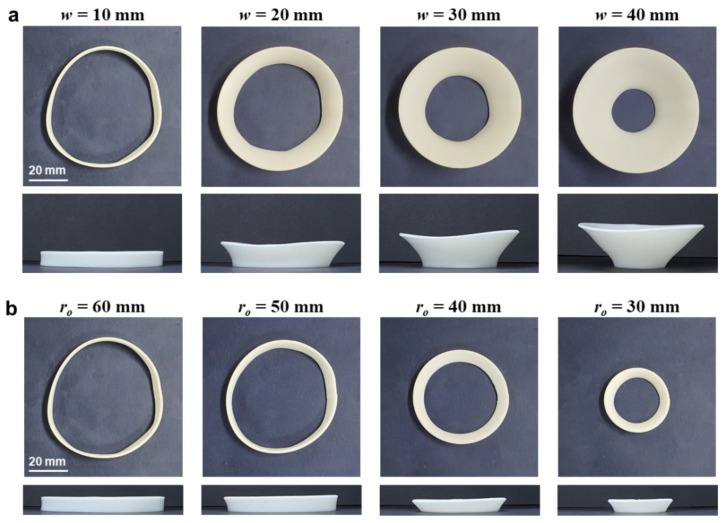
Thermal deformation results of the full annulus frame: (**a**) deformed shapes with various annulus widths (*r_o_ =* 60 mm); (**b**) deformed shapes with various outer radii (*w* = 10 mm). Here, the top and front views are provided to show the three-dimensional shape transformation.

The full annulus was initially designed to have a 60 mm outer radius and 10 mm width, and its design parameters were varied in the same manner as the previous partial annulus cases: 10 to 40 mm for the width (*w*) and 60 to 30 mm for the outer radius (*r_o_*). Figure 5a,b show the deformed shapes of the full annuli with various widths and outer radii, respectively. Overall, these annuli exhibited three-dimensional (3D) shape transformations in which the outer regions moved upward and formed conical shapes. The shape transformation procedure can be observed in Video S1 (Supplementary Materials). These 2D-to-3D shape transformations differ from the 2D-to-2D shape transformations of the partial annuli shown in Figure 4.

This 2D-to-3D shape transformation can be explained by the unique thermal deformation of the circularly printed annulus. With a partial annulus, the central angle after heat treatment (*β*) should be less than the original central angle (π/2), because the magnitude of the outer circumferential strain ($\varepsilon_{\theta }^{o}$) is larger than that of the inner circumferential strain ($\varepsilon_{\theta }^{i}$), as indicated in Table 2 and Table 3. For a full annulus, however, the deformation mechanism is more complicated than that of a partial annulus because a change in the central angle is not allowed. Accordingly, the central angle of a quarter section should be π/2 even after the anisotropic shrinkage, which results in additional shrinkage along the circumferential direction. Moreover, a full annulus undergoes thermal expansion along the radial direction; thus, the deformed width (w*) should be larger than the original width (*w*). This complicated deformation behavior, which includes both radial expansion and circumferential contraction, induces the subsequent 2D-to-3D shape transformations, as shown in Figure 5.

**Figure 6:**
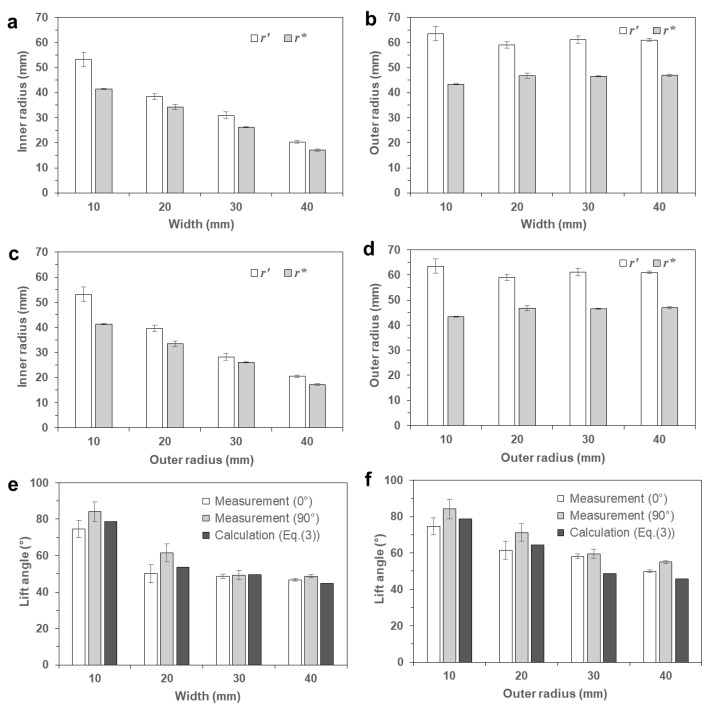
Comparison of deformed shapes of the full annuli: (**a**) deformed inner radii with various widths; (**b**) deformed outer radii with various widths; (**c**) deformed inner radii with various outer radii; (**d**) deformed outer radii with various outer radii; (**e**) lift angles with various widths; (**f**) lift angles with various outer radii.

Figure 6a compares the deformed inner radii of the partial and full annuli, for various widths. The deformed radii of the full annuli (r_i_*) are smaller than those of the partial annuli (*r_i_*′), while their difference (*r_i_*′ − r_i_*) decreases as the width increases. This result can be explained by the constrained circumferential contraction of the full annulus; the outer circumferential strain ($\varepsilon_{\theta }^{o}$) should be the same as the inner circumferential strain ($\varepsilon_{\theta }^{i}$). Figure 6b compares the deformed outer radii with various widths, showing that the size reduction in the outer radius (*r_o_*′ − r_o_*) was larger than that in the inner radius (*r_i_*′ − r_i_*). Accordingly, the amount of radius difference (Δr*) increased as the width increased; thus, the lift angle (*γ*) subsequently decreased, as shown in Figure 5a.

Figure 6c,d compare the deformed inner and outer radii with various outer radii. The overall trends were the same as the previous case (Figure 6a,b), in which the deformed radii of the full annuli (r_i_*) were smaller than those of the partial annuli (*r_i_*′), and the size reduction of the outer radius (*r_o_*′ − r_o_*) was larger than that of the inner radius (*r_i_*′ − r_i_*). As the outer radius increased, the amount of size reduction decreased, whereas the amount of radius difference (Δr*) increased. Therefore, the lift angle (*γ*) subsequently decreased according to the increase in the outer radius, as shown in Figure 5b.

Figure 6e,f compare the lift angles calculated from Equation (3) with the measured values. The angle measurements were conducted from the front view (0°) and the side view (90°) because the deformed annuli were not rotationally symmetric. Although the experimental observations revealed deviations with different viewing angles (i.e., 0° and 90°), the theoretical calculation appropriately predicted the overall trend that the lift angle decreased as the width and outer radius increased. The deviation with different view angles can be attributed to the unstable deformation mechanism of the proposed shape transformation, which is discussed in Section 3.4 in detail.

### 3.3. Numerical Investigation of Thermal Residual Stress

**Figure 7:**
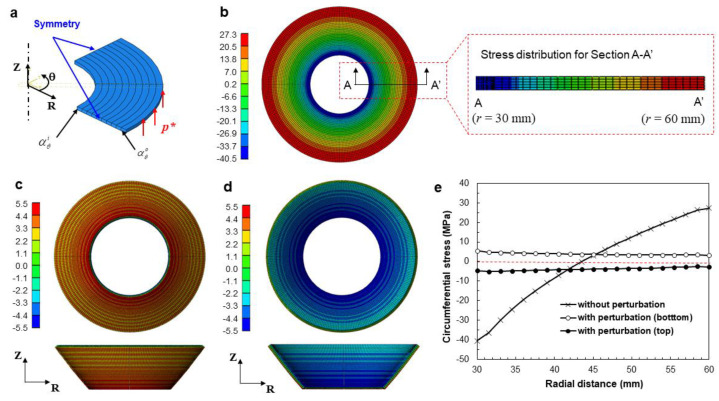
Finite element (FE) analysis results for the residual circumferential stress (unit: MPa): (**a**) analysis model with boundary conditions; (**b**) without perturbation; (**c**) with perturbation (bottom side, the bottom and front views); (**d**) with perturbation (top side, the top and sectional views); (**e**) comparison of the circumferential stress along the path A–A’.

To investigate the thermal residual stress of a full annulus, a thermomechanical finite element (FE) analysis was performed. ABAQUS/Implicit^®^ was used to predict the thermal residual stress of an anisotropically printed annular frame (*r_o_* = 60, *w* = 30 mm). The FE model was constructed using eight-node hexahedral elements. The relevant mesh size was set to 1 mm in the in-plane direction (i.e., the radial and circumferential directions) and 0.32 mm in the thickness direction. Figure 7a shows the FE model of the full annulus, which was prepared for a quarter section by considering geometrical symmetry. To impose the radial and circumferential anisotropy, the coordinate system was converted to a cylindrical coordinate system. The apparent thermal expansion coefficients were then defined by assuming a linear relationship between the measured circumferential strain and the temperature gradient during heat treatment (Δ*T*), as follows [41]:

(6) $$\alpha_{\theta }^{i}=\frac{\varepsilon_{\theta }^{i}}{\Delta T},$$

(7) $$\alpha_{\theta }^{o}=\frac{\varepsilon_{\theta }^{o}}{\Delta T},$$

where $\alpha_{\theta }^{i}$ and $\alpha_{\theta }^{o}$ denote the apparent thermal expansion coefficients along the inner and outer circumferential directions, respectively. Because the circumferential strain and the resulting thermal expansion were not consistent for the inner and outer regions, as shown in Table 1, the circumferential thermal expansion coefficient was assumed to be a linear interpolation function of the radius (*r*), as follows:

(8) $$\alpha_{\theta }(r)=\frac{r_{o}-r}{r_{o}-r_{i}}\alpha_{\theta }^{i}+\frac{r-r_{i}}{r_{o}-r_{i}}\alpha_{\theta }^{o}.$$

The resulting circumferential thermal expansion coefficients were then calculated from Equations (6) and (7), and the resulting values are given in Table 4. To allocate the linearly varying thermal expansion coefficients, the analysis domain was divided into 10 subdomains along the radial direction, as shown in Figure 7a. The thermal expansion coefficient for each subdomain was calculated from Equation (5) and was allocated for each subdomain. The thermal expansion coefficients along the radial and thickness directions and elastic modulus were taken from Table 1. The initial and ambient temperatures were set to 22 and 150 °C, respectively. A linear static analysis was carried out for a quarter region with symmetric boundary conditions.

Figure 7b shows the deformed shape and distribution of the circumferential stress (*σ_θ_*) of the full annulus. The residual circumferential stress showed a large deviation depending on the radial position (−40.5–27.3 MPa), revealing tensile residual stress in the outer region and compressive residual stress in the inner region. This can be explained by the opposite thermal strains along the circumferential and radial directions, as well as by the difference in the circumferential strains along the inner and outer boundary surfaces, as discussed in Section 3.2. However, the deformed shape was still a 2D shape, and the resulting stress also showed 2D distribution. Moreover, the stress distributions of the top and bottom surfaces were almost the same, as illustrated in the sectional stress distribution (A–A’).

To simulate the 2D-to-3D shape transformation, the thermal deformation analysis needs to include structural instability. To consider structural instability by initiating out-of-plane deformation, a small perturbation pressure (p* = 20 kPa) was temporarily applied vertically on the outer surface, as shown in Figure 7a. Then the perturbation pressure was removed and the thermal deformation analysis continued. Figure 7c shows the resulting out-of-plane deformation of the annular frame from the bottom and front views, which exhibits a similar deformation mode to the experimental results in Figure 5a. The bottom face was under tensile residual stress, in the range between 3.15 and 5.49 MPa. In contrast, the top face was under compressive residual stress, in the range between −4.68 and −2.81 MPa, as shown in Figure 7d.

Figure 7e compares the resulting circumferential stress profiles of the three cases, along the path A–A’. It was observed that the consideration of the out-of-plane perturbation resulted in a significant reduction in residual stress. The resulting residual stress shows uniform distributions along the radial direction and a small amount of stress gradient along the thickness direction. This stress relaxation in accordance with the out-of-plane perturbation then explains the 2D-to-3D shape transformation of the full annulus with circumferential anisotropy.

### 3.4. Effect of Constrained Heat Treatment

**Figure 8:**
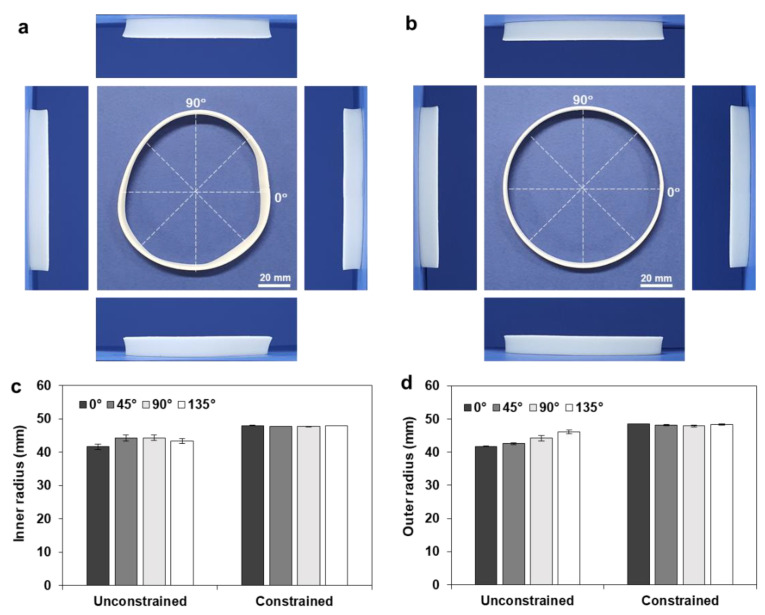
Comparison of the roundness of the deformed annulus (*r_o_* = 60, *w* = 10 mm): (**a**) unconstrained deformation; (**b**) constrained deformation; (**c**) comparison of the inner radius (r_i_*); (**d**) comparison of the outer radius (r_o_*).

Although the circumferentially printed full annuli showed 2D-to-3D shape transformation, their deformed shapes were not uniform; the deformed annulus cannot be regarded as a circular annulus, as illustrated in Figure 8a. Moreover, four side views exhibited different shapes and lift angles (*γ*).

To obtain a uniform shape transformation, heat treatment was conducted with the aid of a geometric constraint, by locating a deformation guide inside the annulus. Here, the deformation guide was fabricated with a 96 mm tip diameter and a 90° draft angle. Figure 8b shows the deformed shape of the full annulus (*r_o_* = 60, *w* = 10 mm), which shows uniform shape transformation with a circular shape. To compare the circularity of the deformed annulus quantitatively, the inner and outer radii of the free and constrained samples were compared, as shown in Figure 8c,d. The inner and outer radii of three samples were measured along the four directions (0°, 45°, 90°, and 135°), and the resulting ranges of the inner and outer radii were found to be 41.63–44.29 and 41.75–46.08 mm, respectively. The constrained heat treatment resulted in uniform dimensions with negligible deviations in the inner (47.68–47.99 mm) and outer (47.85–48.50 mm) radii, while the unconstrained heat treatment showed large deviations and a resulting deterioration in circularity.

**Figure 9:**
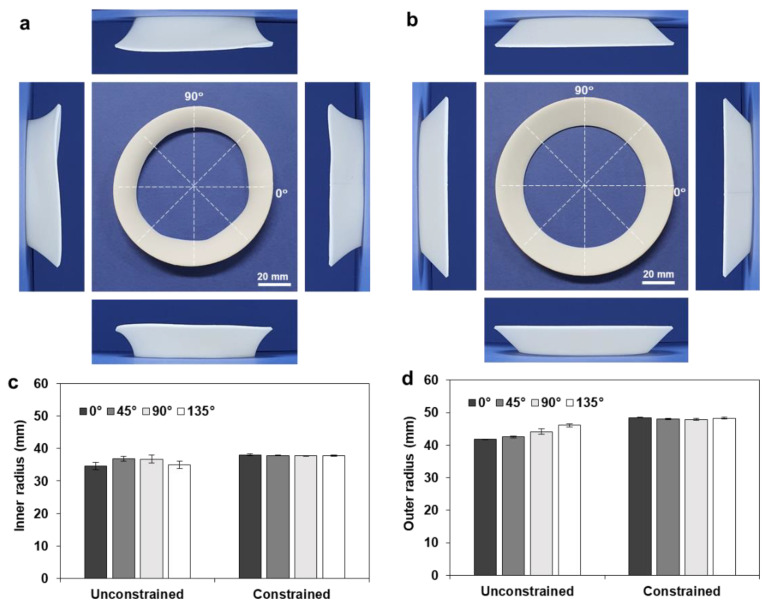
Comparison of the roundness of the deformed annulus (*r_o_* = 60, *w* = 20 mm): (**a**) unconstrained deformation; (**b**) constrained deformation; (**c**) comparison of the inner radius (r_i_*); (**d**) comparison of the outer radius (r_o_*).

Figure 9a,b show the deformed shapes of the full annulus (*r_o_* = 60, *w* = 20 mm) after unconstrained and constrained heat treatments, respectively. Here, the deformation guide was fabricated with a 76 mm tip diameter and a 45° drift angle. The shape transformation during the constrained heat treatment can be observed in Video S2 (Supplementary Materials). The constrained heat treatment showed a circular shape and uniform lift angles from the four sides, while the unconstrained heat treatment exhibited noncircular deformation and different side views. Figure 9c,d compare the resulting inner and outer radii of the unconstrained and constrained samples. 

**Figure 10:**
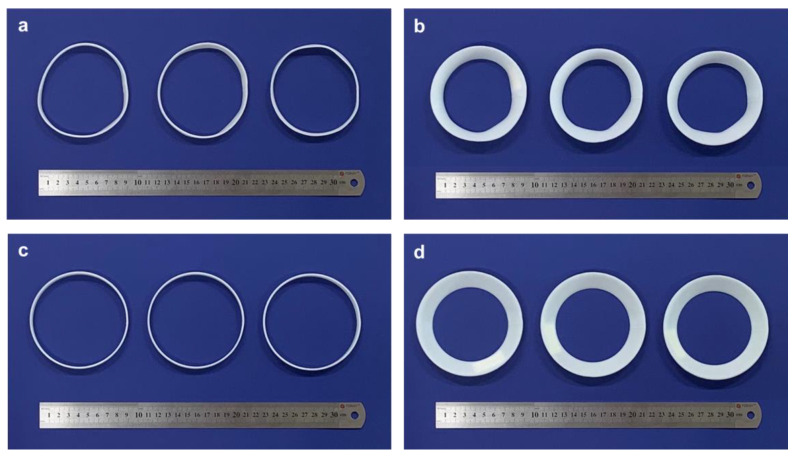
Comparison deformation repeatability: (**a**) unconstrained deformation (*w* = 10 mm); (**b**) unconstrained deformation (*w* = 20 mm); (**c**) constrained deformation (*w* = 10 mm); (**d**) constrained deformation (*w* = 20 mm).

Figure 10a,b show deformed samples after unconstrained heat treatments, with different annulus widths of 10 and 20 mm, respectively. The deformed samples were not uniform in shape and showed different shape transformations among the three samples. These varying results indicate that the current shape transformation is not repeatable in terms of ensuring consistent dimensional accuracy. In contrast, after the constrained heat treatment, all the deformed samples retained almost the same shapes, as shown in Figure 10c,d, thus achieving outstanding repeatability in shape transformation. These results confirm that the constrained heat treatment ensures a more uniform and repeatable shape transformation than the unconstrained heat treatment, which is advantageous for industrial manufacturing applications.

### 3.5. Shape Transformation of Annular Composite Structures

#### 3.5.1. Additive Manufacturing of Annular Composite Structures

The developed shape transformation process was then applied to fabricate a composite frame–membrane structure. Here, a thin membrane was inserted inside the additively manufactured annular frame. The membrane material was a 100% polyester fabric, whose elongation and melting temperatures were 20% and 270 °C, respectively. This polyester fabric was prepared with a size of 160 × 160 × 0.2 mm^3^ and was inserted during the AM procedure. To fabricate this kind of frame–membrane composite structure, the upper and lower frames should be fabricated first using a traditional manufacturing process such as injection molding and should then be bonded with the membrane.

**Figure 11:**
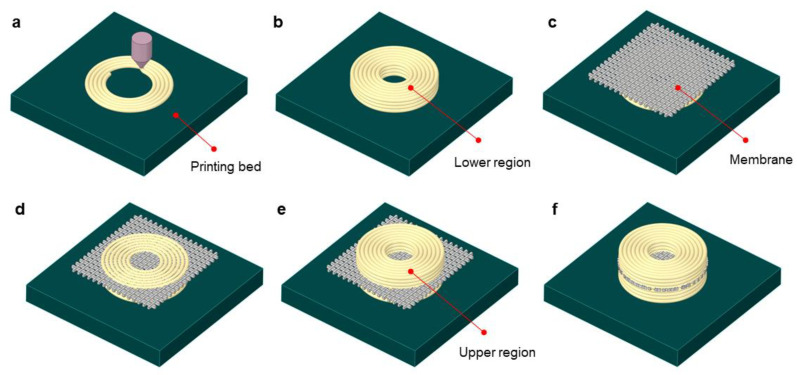
Additive manufacturing (AM) procedure for fabricating the annular composite structure: (**a**) printing of the first layer; (**b**) building of the lower region; (**c**) installation of a thin membrane; (**d**) printing of the overlaid layer; (**e**) building of the upper region; (**f**) trimming of the membrane.

Figure 10 shows the in situ AM and assembly procedure of the membrane–frame composite structure. A number of annular layers were printed along the circular path using the ME type AM process (Figure 11a), and the lower region was built as illustrated in Figure 11b. A rectangular membrane was placed on the top of the lower region, as shown in Figure 11c. The AM process was then started again for the next layer (Figure 11d), and this sequence was repeated to build a number of layers to construct the upper region (Figure 11e). The exterior membrane was then trimmed out, as shown in Figure 11f, thus fabricating a membrane–frame composite structure without an additional assembly or bonding procedure.

**Figure 12:**
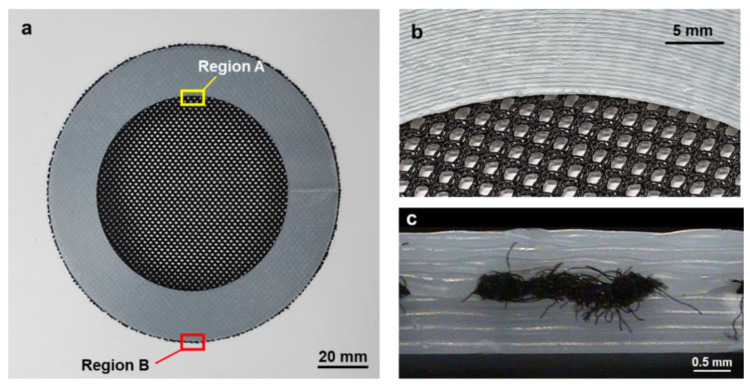
(**a**) Fabricated annular composite structure; (**b**) enlarged image of *Region A* (top view); (**c**) enlarged image of *Region B* (side view).

Figure 12a shows the fabricated annular composite after trimming. An enlarged image of *Region A* is illustrated in Figure 12b, in which a number of circular printing paths for the upper region can be observed. Figure 11c is an enlarged image of *Region B* from the side view, which shows that the fabric was well fused between the upper and lower printed regions. Moreover, it shows that the first layer of the upper region infiltrated into the void regions of the fabric and adhered to the last layer of the lower region. Accordingly, these three components were strongly integrated without an additional assembly procedure.

#### 3.5.2. Shape Transformation of the Annular Composite Structures

**Figure 13:**
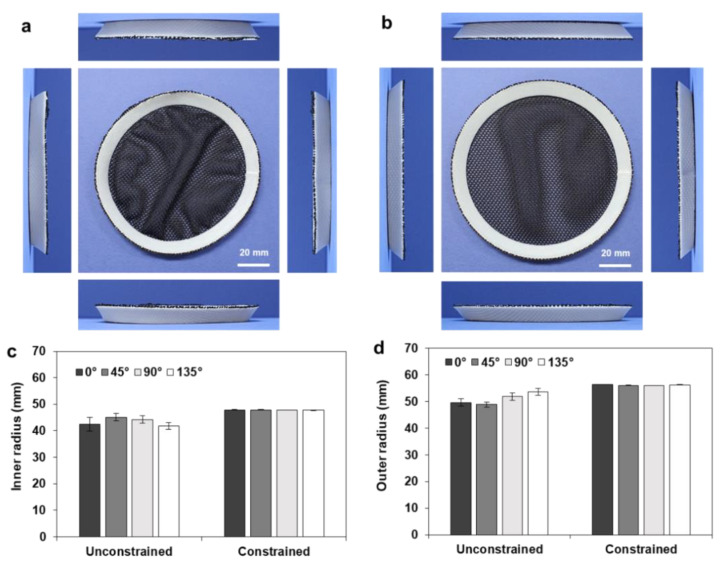
Deformed shape of the composite frame (*r* = 60, *w* = 10 mm); (**a**) unconstrained deformation; (**b**) constrained deformation; (**c**) comparison of the inner radius (r_i_*); (**d**) comparison of the outer radius (r_o_*).

Figure 13a shows the deformed shape of the fabricated annular composite (*r_o_* = 60, *w* = 10 mm) after unconstrained heat treatment, which cannot be regarded as a circular annulus. The inner and outer radii of the deformed annulus were measured along the four directions (0°, 45°, 90°, and 135°), and the measured results are compared in Figure 13c,d. The range of the inner radius was 41.88–45.09 mm, and that of the outer radius was 48.93–53.67 mm. These values were larger than those of the pure annular disc shown in Figure 8, which were 41.63–44.29 mm for the inner radius and 47.85–48.50 mm for the outer radius. This reduced shrinkage can be attributed to the deformation resistance of the polyester fabric. Figure 13b shows the deformed shape after the constrained heat treatment, where the deformation guide was prepared with a 96 mm tip diameter and a 40° draft angle considering the results of Figure 13c. As compared in Figure 13d, the resulting deviations in the inner and outer radii were significantly reduced; the range of the inner radius was 47.76–47.83 mm, and that of the outer radius was 55.99–56.40 mm.

**Figure 14:**
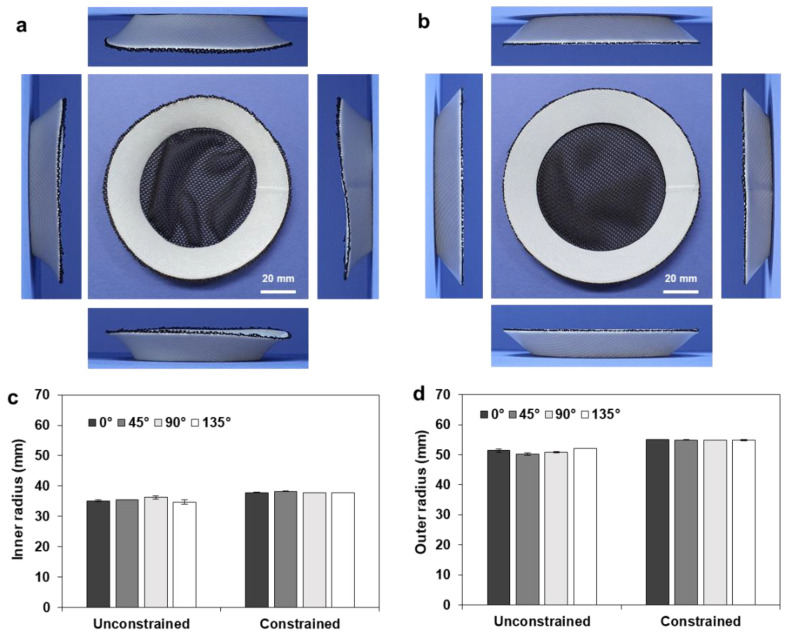
Deformed shape of the composite frame (*r* = 60, *w* = 20 mm): (**a**) unconstrained deformation; (**b**) constrained deformation; (**c**) comparison of the inner radius (r_i_*); (**d**) comparison of the outer radius (r_o_*).

Figure 14a,b show the deformed shapes of another annular composite (*r_o_* = 60, *w* = 20 mm) after the unconstrained and constrained heat treatments, respectively. The corresponding shape transformations during the unconstrained and constrained heat treatments are provided in Videos S3 and S4 (Supplementary Materials), respectively. In the case of the constrained heat treatment, a deformation guide was prepared with a 76 mm diameter and a 50° draft angle. Figure 14c,d compare the relevant inner and outer radii of the deformed annuli. The unconstrained heat treatment also showed nonuniform deformation, with large deviations in the inner radius (34.53–36.79 mm) and outer radius (41.75–46.08 mm). In contrast, the constrained heat treatment showed uniform deformation with significantly reduced deviations in the inner radius (37.73–38.03 mm) and outer radius (47.85–48.50 mm). These results indicate that the constrained heat treatment also ensures uniform and repeatable shape transformation in frame–membrane composite structures.

## 4. Conclusions

In this study, a cost-effective and reliable shape transformation method was developed for producing annular frame structures. To reduce the processing cost, the proposed method used an ME type 3D printer and a plain thermoplastic filament (ABS) without a shape memory function. For the subsequent shape transformation of this material, the printing path for an annular disc was programmed along a circular direction to induce circumferential anisotropy. The additional heat treatment at 150 °C resulted in the 2D-to-3D shape transformation of the printed discs. This 3D deformation mechanism was further analyzed by thermomechanical FE analysis, which demonstrated that the 2D-to-3D shape transformations originated with the relaxation of residual stress introduced by the circumferential anisotropy. The use of a personal ME printer and an electric oven for this process demonstrates the cost-effectiveness of this shape transformation approach compared with other 4D printing methods that use smart materials or multi-material 3D printers.

To improve dimensional accuracy and repeatability, the proposed shape transformation was conducted using an auxiliary geometric constraint. The constraint ensured that the 2D annular discs were transformed to 3D annular frames with higher dimensional accuracy and circularity, compared with the unconstrained deformation. Furthermore, the constrained heat treatment ensured more uniform and repeatable shape transformations than the unconstrained heat treatment, which is advantageous for industrial manufacturing applications. The proposed shape transformation with a geometric constraint was then further applied to the AM and in situ assembly of a composite frame–membrane structure. A functional membrane was integrated within a 3D annular frame without additional assembly or bonding procedures. However, the constrained transformation may be limited by the shape of the deformation guide, which must be removed after heat treatment.

Unlike conventional 4D printing methods, the proposed approach enables an irreversible and precise shape transformation. Therefore, it can be utilized for the fabrication of various 3D frame-and-membrane composite structures that require a series of manufacturing and assembly processes with permanent deformations. For example, filtering devices with curved shapes can be manufactured using the proposed in situ assembly and shape transformation processes, by integrating the shaping (i.e., injection molding of the curved frame) and the bonding processes; thus, manufacturing costs can be reduced. According to these advantages, a further study to develop a wearable device that integrates thin conductive circuits in a nonconductive 3D frame will be performed in the near future.

## Figures and Tables

**Table 1:** Directional elastic moduli (*E*), tensile strengths (*σ_u_*), and apparent thermal expansion coefficients of additively manufactured specimens.

Direction	*E* (GPa)	*σ_u_* (MPa)	α*_l_* (×10^−3^/°C)	α*_l_* (×10^−3^/°C)
Length (*x*)	2.178	28.47	−1.53	0.16
Width (*y*)	2.497	33.47	0.95	−0.40
Height (*z*)	2.258	8.39	2.22	2.22

**Table 2:** Comparison of thermal strains with various annulus widths (*w*).

*r_o_* (mm)	60	50	40	30
$\varepsilon_{\theta }^{o}$	−0.155 ± 0.002	−0.161 ± 0.005	−0.161 ± 0.016	−0.166 ± 0.007
$\varepsilon_{\theta }^{i}$	−0.145 ± 0.003	−0.147 ± 0.009	−0.151 ± 0.010	−0.140 ± 0.002
$\varepsilon_{r}$	0.0410 ± 0.004	0.0430 ± 0.009	0.0405 ± 0.010	0.0424 ± 0.005
$\varepsilon_{z}$	0.135 ± 0.005	0.133 ± 0.009	0.129 ± 0.006	0.135 ± 0.005

**Table 3:** Comparison of thermal strains with various outer radii (*r_o_*).

*r_o_* (mm)	60	50	40	30
$\varepsilon_{\theta }^{o}$	–0.155 ± 0.002	–0.158 ± 0.005	–0.165 ± 0.004	–0.177 ± 0.004
$\varepsilon_{\theta }^{i}$	–0.145 ± 0.003	–0.144 ± 0.002	–0.151 ± 0.010	–0.132 ± 0.005
$\varepsilon_{r}$	0.0410 ± 0.004	0.0441 ± 0.002	0.0454 ± 0.002	0.0445 ± 0.006
$\varepsilon_{z}$	0.135 ± 0.005	0.136 ± 0.006	0.139 ± 0.004	0.135 ± 0.005

**Table 4:** Directional thermal expansion coefficients and the relevant simulation conditions.

Simulation Conditions	Contents
Circumferential thermal expansion ($\alpha_{\theta }^{o}$)	−2.07 × 10^−3^
Circumferential thermal expansion ($\alpha_{\theta }^{i}$)	−1.53 × 10^−3^
Radial thermal expansion ($\alpha_{r}$)	0.95 × 10^−3^
Axial thermal expansion ($\alpha_{z}$)	2.22 × 10^−3^
Initial temperature (°C)	22
Ambient temperature (°C)	150
Perturbation pressure (kPa)	20

## Data Availability

Data sharing is not applicable to this article.

## Supplementary Materials

The following are available online at https://www.mdpi.com/1996-1944/14/6/1383/s1: Video S1. Unconstrained shape transformation of the circularly printed annular frame (*r_o_* = 60, *w* = 20 mm); Video S2. Constrained shape transformation of the circularly printed annular frame (*r_o_* = 60, *w* = 20 mm); Video S3. Unconstrained shape transformation of the annular composite structure (*r_o_* = 60, *w* = 20 mm); Video S4. Constrained shape transformation of the annular composite structure (*r_o_* = 60, *w* = 20 mm).
